# Targeting angiogenesis in multiple myeloma by the VEGF and HGF blocking DARPin^®^ protein MP0250: a preclinical study

**DOI:** 10.18632/oncotarget.24351

**Published:** 2018-01-30

**Authors:** Luigia Rao, Kim De Veirman, Donato Giannico, Ilaria Saltarella, Vanessa Desantis, Maria Antonia Frassanito, Antonio Giovanni Solimando, Domenico Ribatti, Marcella Prete, Andreas Harstrick, Ulrike Fiedler, Hendrik De Raeve, Vito Racanelli, Karin Vanderkerken, Angelo Vacca

**Affiliations:** ^1^ Department of Biomedical Sciences and Human Oncology, Unit of Internal Medicine and Clinical Oncology, University of Bari Medical School, Bari, Italy; ^2^ Department of Hematology and Immunology, Myeloma Center Brussels, Vrije Universiteit Brussel, Brussels, Belgium; ^3^ Department of Basic Medical Sciences, Neurosciences, and Sensory Organs, Section of Human Anatomy and Histology, University of Bari Medical School, National Cancer Institute "Giovanni Paolo II", Bari, Italy; ^4^ Molecular Partners AG, Zürich-Schlieren, Switzerland; ^5^ Department of Pathology, UZ Brussel, Vrije Universiteit Brussel, Brussels, Belgium

**Keywords:** multiple myeloma, angiogenesis, DARPin, dual inhibitor, syngeneic mouse model

## Abstract

The investigational drug MP0250 is a multi-specific DARPin^®^ molecule that simultaneously binds and neutralizes VEGF and HGF with high specificity and affinity. Here we studied the antiangiogenic effects of the MP0250 in multiple myeloma (MM). In endothelial cells (EC) isolated from bone marrow (BM) of MM patients (MMEC) MP0250 reduces VEGFR2 and cMet phosphorylation and affects their downstream signaling cascades. MP0250 influences the secretory profile of MMEC and inhibits their in vitro angiogenic activities (spontaneous and chemotactic migration, adhesion, spreading and capillarogenesis). Compared to anti-VEGF or anti-HGF neutralizing mAbs, MP0250 strongly reduces capillary network formation and vessel-sprouting in a Matrigel angiogenesis assay. MP0250 potentiates the effect of bortezomib in the same in vitro setting. It significantly reduces the number of newly formed vessels in the choriollantoic membrane assay (CAM) and the Matrigel plug assay. In the syngeneic 5T33MM tumor model, MP0250 decreases the microvessel density (MVD) and the combination MP0250/bortezomib lowers the percentage of idiotype positive cells and the serum levels of M-protein. Overall results define MP0250 as a strong antiangiogenic agent with potential as a novel combination drug for treatment of MM patients.

## INTRODUCTION

Multiple Myeloma (MM) is a B cell neoplasia which is characterized by clonal plasma cell (PC) expansion in the bone marrow (BM) and serious clinical manifestations such as osteolytic bone lesions, anemia, hypercalcemia and renal failure [1]. Disease progression correlates with increased microvessel density (MVD) in the BM which is a prognostic factor [2]. Changes in tumor BM microenvironment lead to an impaired balance between pro- and antiangiogenic factors which induces angiogenesis [3]. First of all we have demonstrated the angiogenic potential of PCs isolated from the BM of MM patients [4] after that we described the angiogenic phenotype of BM-derived endothelial cells (EC) of MM patients (MMEC) [5].

Among growth factors responsible for MM-associated angiogenesis, vascular endothelial growth factor (VEGF) and hepatocyte growth factor (HGF) are recognized as key angiogenic factors [6]. VEGF stimulates vascular permeability, EC migration, proliferation and survival through the activation of the RAS/RAF/ERK/MAPK pathways [7]. The HGF/cMet axis is constitutively activated in MMEC and mediates angiogenic functions such as migration and tube formation [8]. HGF exerts an angiogenic effect by increasing the activation of the VEGF/VEGFR2 pathway and by the downregulation of thrombospondin [9].

Altogether these data indicate that angiogenesis is a key driver of MM malignancy and an attractive target for MM treatment. Several antiangiogenic strategies have been developed and investigated; among these are (i) drugs targeting both MM and neovessels such as the immunomodulatory drugs (IMiDs) and the proteasome inhibitors bortezomib and carfilzomib [10–12], along with (ii) drugs that interfere with specific EC functions by altering cell signaling pathways activating angiogenesis such as bevacizumab/Avastin [7]. Avastin^®^, a humanized mAb, was the first FDA approved angiogenesis inhibitor able to neutralize VEGF and prevent its binding to VEGF receptors on EC [13, 14].

Advances in protein engineering have allowed the development of new binding proteins able to overcome several limitations of the drugs commonly used in clinical practice. Designed ankyrin repeat proteins (DARPin^®^) are among the promising non-immunoglobulin binding proteins. DARPin^®^ molecules are high-affinity binding proteins with high target specificity and high biophysical stability [15]. MP0250 is a multi-domain DARPin^®^ drug candidate with binding specificities for VEGF-A (the main isoform of VEGF), HGF and human serum albumin and it is the first multi-functional DARPin^®^ protein in clinical trials. It binds simultaneously VEGF and HGF, hence preventing the interaction with VEGFR2 and cMet respectively [16, 17]. MP0250 also exerts potent inhibition of the separate functions of VEGF and HGF in VEGF- and HGF-dependent cell lines, as well as in mouse xenografted models [18].

Given that VEGF and HGF play a critical role in MM [8, 19], we investigated the antiangiogenic activity exerted by MP0250 on MMEC. We demonstrated *in vitro* that MP0250 inhibits activation of MMECs by reduction of VEGFR2 and cMet phosphorylation and modulation of their downstream signaling. *In vivo,* MP0250 shows antiangiogenic activity in a chick chorioallantoic membrane (CAM) assay, a Matrigel plug assay, and in a 5T33MM syngeneic MM mouse model.

## RESULTS

### MP0250 influences VEGFR2 and cMet phosphorylation in MMEC

**Figure 1:**
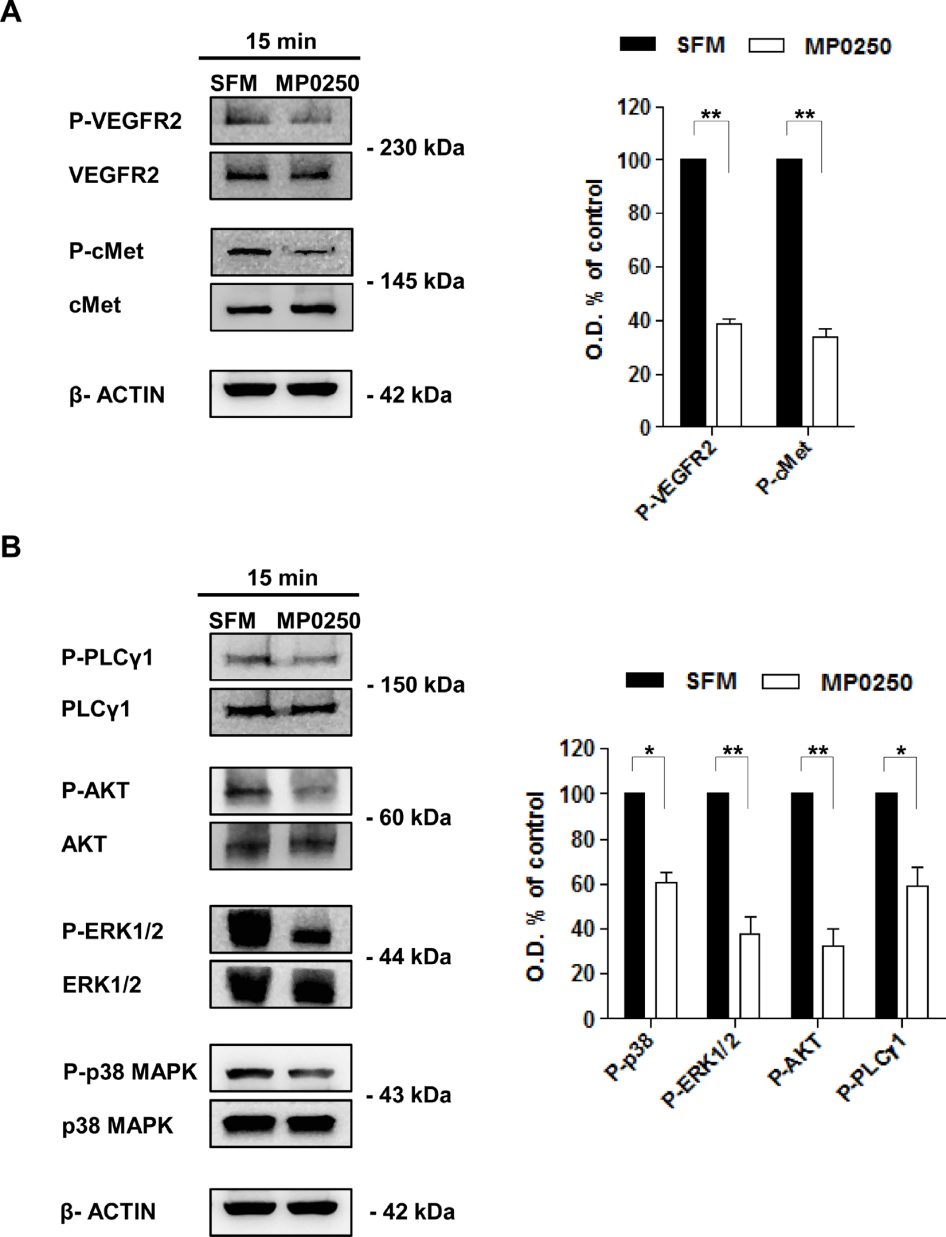
MP0250 affects VEGFR2 and cMet phosphorylation and their downstream signaling (**A**) Levels of p-VEGFR2 (Tyr1175) and p-cMet (Tyr1234/1235) were evaluated by immunoblot after 15 minutes of MP0250 treatment. MMEC cultured in SFM are the control. Graph shows the results of six independent experiments. (**B**) Phosphorylation levels of AKT (Ser473), PLCγ1 (Ser1248), P38 (Thr180/Tyr182) and ERK1/2 (Thr202/Tyr202) are analyzed. Densitometric analysis is shown. Values are expressed as mean of six independent experiments ± SD, ^*^P < 0.05; and ^**^P < 0.01 versus SFM as control.

As MP0250 binds simultaneously to VEGF and HGF preventing interaction with their receptors, we tested whether MP0250 impacted the activation of VEGFR2 and cMet in MMEC. In accord with our previous studies [8, 19], immunoblot analysis showed that MMEC had high levels of phosphorylated (p)-VEGFR2 and p-cMet (Figure 1A) mediated by autocrine stimulation by VEGF and HGF expression. A concentration-dependent reduction of p-VEGFR2 and p-cMet expression was observed by cytofluorimetric analysis after treatment of MMEC with increasing doses of MP0250 (0.4 to 3.2 µM) (Supplementary Figure 1A) but no effect on cell viability (Supplementary Figure 1B) or induction of apoptosis were seen (Supplementary Figure 1C). Immunoblot analysis showed that treatment of MMEC with MP0250 for 15 minutes significantly reduced p-VEGFR2 and p-cMet levels (Figure 1A) with parallel inhibition of p-AKT, p-ERK1/2, p-p38 MAPK and p-PLCγ1 (Figure 1B). Similar results were obtained after 12 hours of treatment (Supplementary Figure 2). These data suggest that MP0250 exerts a rapid and persistent effect on the VEGFR2 and cMet signaling pathways.

### MP0250 inhibits *in vitro* MMEC functions involved in angiogenesis

**Figure 2:**
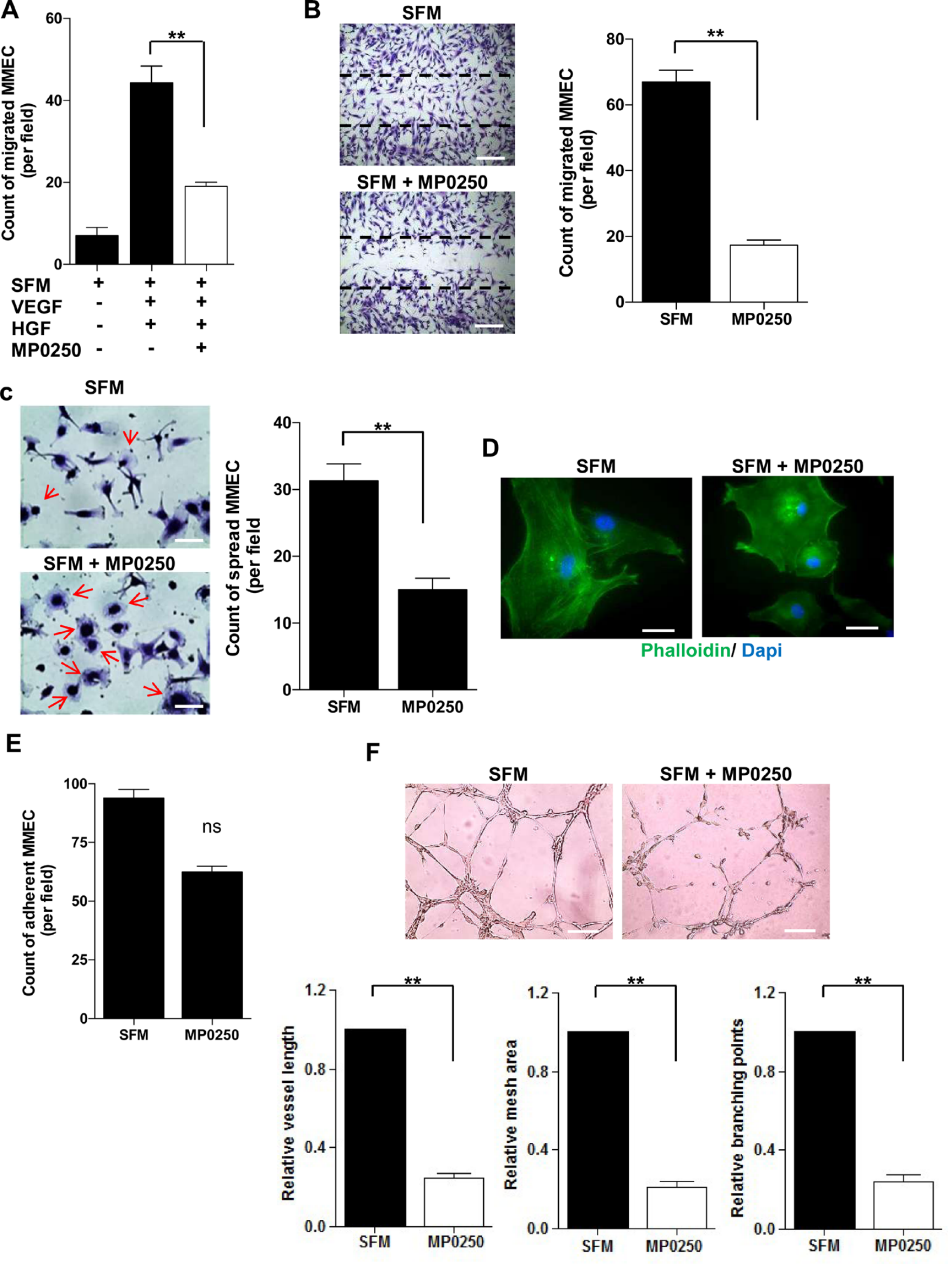
*In vitro* effects of MP0250 on MMEC functions (**A**) MMEC chemotaxis was evaluated versus SFM or SFM plus VEGF and HGF (50 ng/mL each one) with or without MP0250. Bars represent mean number of migrated MMEC in five X200 fields per patient. (**B**) MP0250-treated (2 µM) and SFM-treated MMEC were wounded and number of migrated cells was reported. Pictures of one representative experiment are shown. Original magnification X200. Scale bar = 50 μm. (**C**) MP0250-treated (2 µM) and SFM-treated MMEC were plated on fibronectin. The number of spread cells was determined. (**D**) Immunofluorescence of phalloidin distribution in MMEC treated with 2 µM MP0250 versus SFM. Original magnification X400. Scale bar = 25 μm. (**E**) Adhesion on fibronectin of MP0250-treated (2 µM) and SFM-treated MMEC was evaluated. The number of adherent MMEC is reported. (**F**) MMEC were seeded on Matrigel in SFM with or without 2 µM MP0250. Pictures of one representative experiment are shown. Vessel length, mesh areas and branching points are measured. Original magnification X200. Scale bar = 50 µm. Values are expressed as mean ± SD of ten independent experiments; NS = not significant; ^*^P < 0.05; and ^**^P < 0.01 versus SFM as control.

Next, we investigated whether MP0250 affected MMEC angiogenic functions *in vitro*. MP0250 treatment of MMEC reduced chemotaxis towards VEGF and HGF (Figure 2A) and spontaneous migration (Figure 2B). Cells treated with MP0250 and plated on a fibronectin-coated surface hold a round morphology without spike-like protrusions (red arrows), implying a low spreading capacity (Figure 2C). This was further confirmed by immunofluorescence staining with phalloidin, implying an indirect effect on cytoskeleton reorganization (Figure 2D). Only a marginal effect on MMEC adhesion was observed (Figure 2E). Considering that all the evaluated MMEC functions cooperate to give rise to a tubular morphogenesis *in vitro*, we performed a Matrigel assay: MP0250 treatment resulted in a poor network formation with loss of cell connections and significant decrease in vessel length and areas (Figure 2F). Results suggest that MP0250 exerts *in vitro* a strong antiangiogenic effect impairing main endothelial cell functions.

### MP0250 is more effective than single VEGF- and HGF-neutralizing mAbs

**Figure 3:**
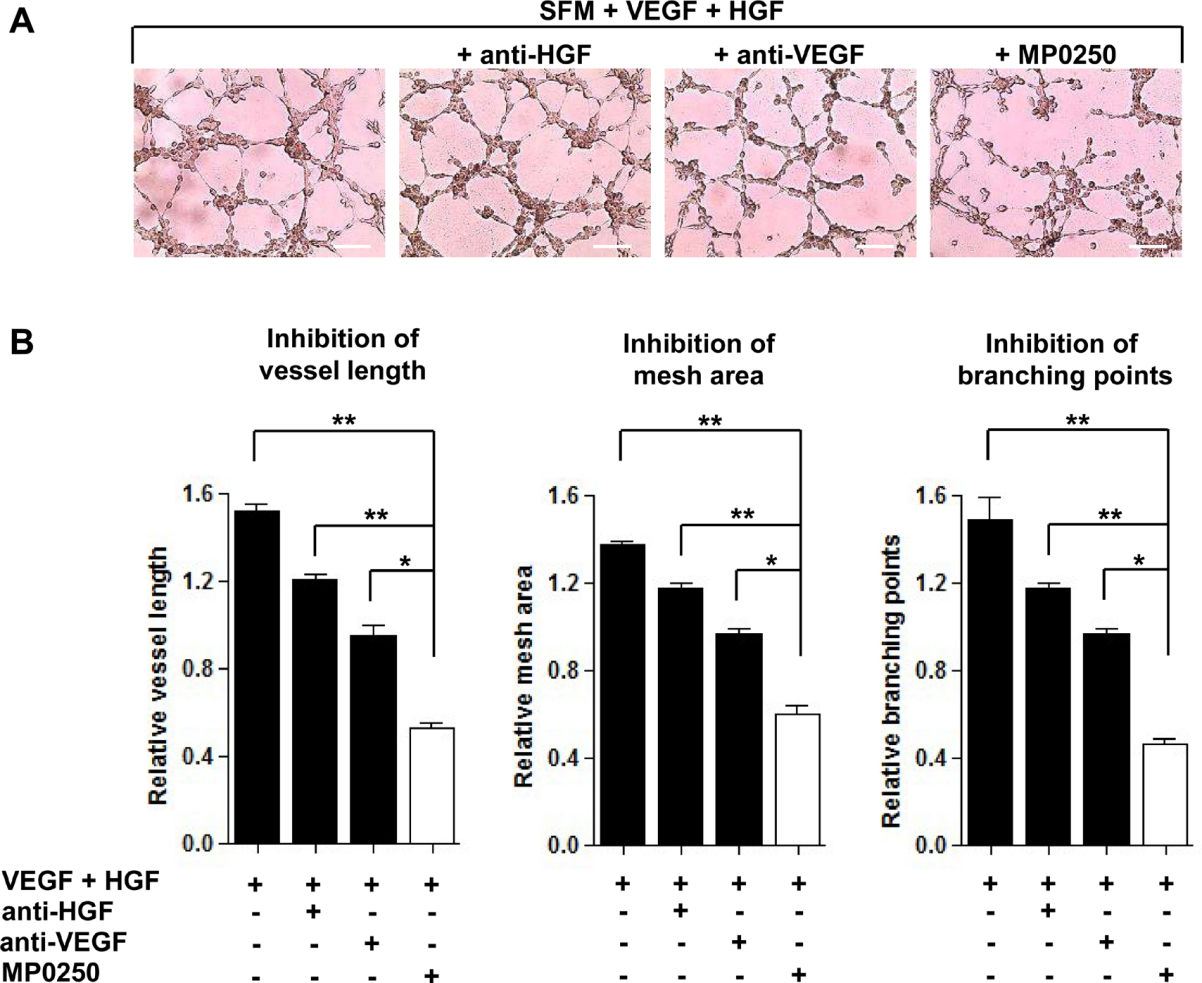
Comparison between MP0250 and single VEGF and HGF neutralizing mAbs in angiogenesis inhibition (**A**) MMEC were seeded on Matrigel in SFM alone or supplemented with MP0250 (2 µM), or anti-VEGF (0.06 μg/mL), or anti-HGF (0.2 μg/mL) mAbs. Pictures of one representative experiment are shown. Original magnification X200. Scale bar = 50 μm. (**B**) Vessel length, mesh areas and branching points are measured. Data are reported as mean ± SD of ten independent experiments; ^*^P < 0.05; and ^**^P < 0.01.

As previously demonstrated [8, 19], MMEC secrete VEGF and HGF and their neutralization impairs *in vitro* angiogenesis. However, several cell types release VEGF and HGF in the BM microenvironment that potentiate the autocrine stimulation of MMEC and contribute to their peculiar over-angiogenic phenotype [4]. To mimic more closely the MM BM microenvironment, we added exogenous VEGF and HGF to serum free medium (SFM) on *in vitro* Matrigel assay. This assessed that MP0250 was more effective in inhibiting angiogenesis than blockade of the individual growth factors with mAbs. MMEC exposed to VEGF and HGF gave rise to a closely-knit vessel network. Addition of anti-VEGF or anti-HGF neutralizing/blocking mAbs to the stimulating medium slightly impacted the vessel organization. In contrast, MP0250 caused a strong inhibition of vessel formation, reducing the number of anastomosed tubes and almost totally preventing cell junction formation, resulting in isolated small cell clumps (Figure 3A). That effect was quantified by measuring vessel length, mesh areas and number of branching points (Figure 3B). Data suggest that by binding both VEGF and HGF, MP0250 exerts a more pronounced inhibition on MMEC angiogenesis than blocking only the individual pathways.

### MP0250 abrogates *in vivo* angiogenesis

**Figure 4:**
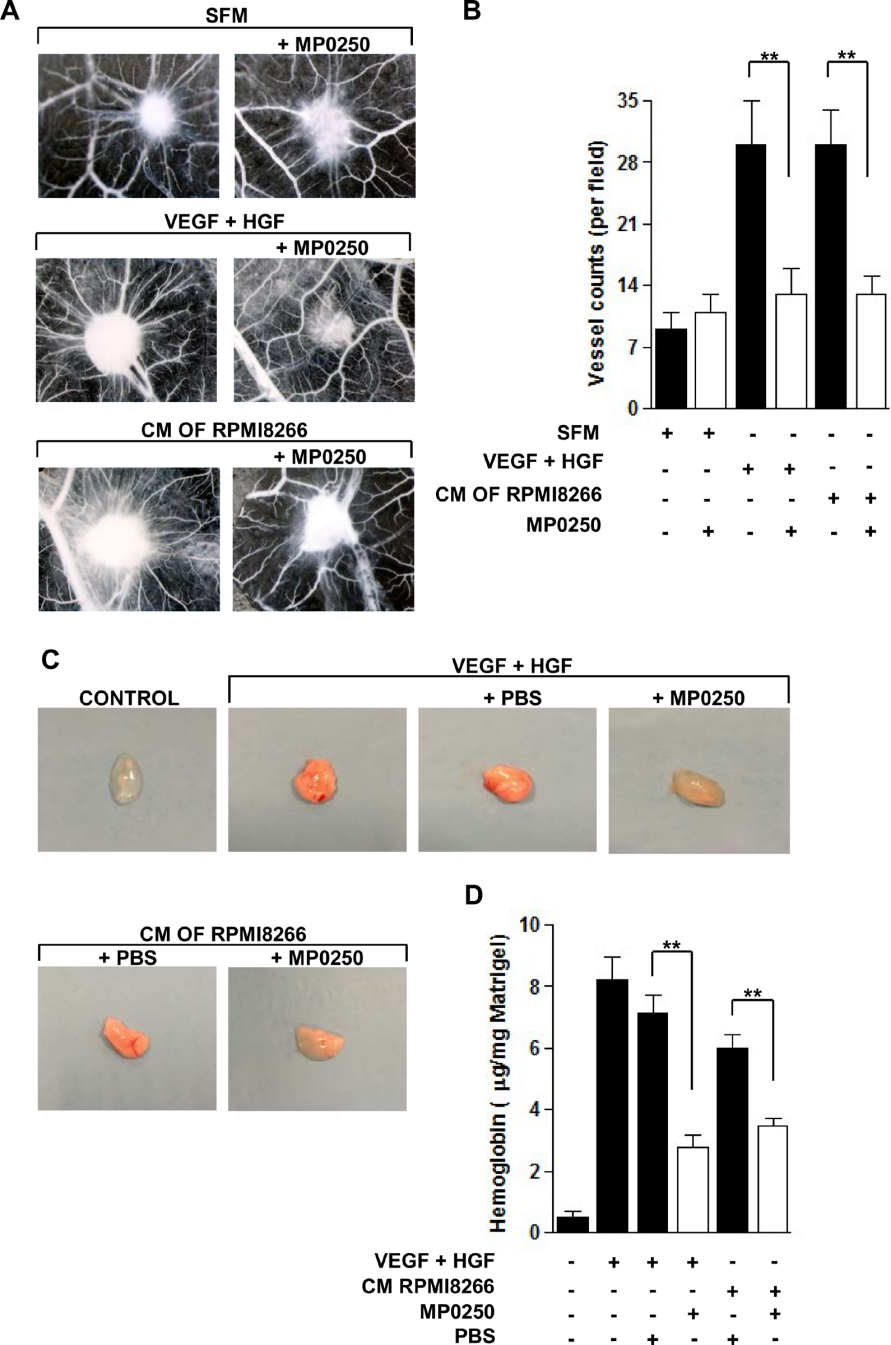
MP0250 inhibits angiogenesis *in vivo* (**A**) CAM assay was performed loading the gelatin sponge with SFM alone or added with VEGF and HGF or with RPMI8266 CM, with or without treatment of MP0250 (20 µM). On day 12, pictures were taken *in ovo*. One representative experiment is shown. Original magnification X50. (**B**) Newly formed vessels were counted. Results were reported as mean ± SD of three independent experiments; ^**^P < 0.01 versus untreated controls. (**C**) NOD/SCID mice were injected subcutaneously with Matrigel with or without VEGF and HGF (200 ng/mL each one) or with RPMI8266 cell CM. MP0250 (4 mg/kg) or PBS (its vehicle) were administered intraperitoneally every 72 hours, 4 times. Pictures of a representative experiment of plugs are shown. (**D**) Hemoglobin content of each plug was measured using Drabkin’s assay and normalized to its weight. Values are expressed as mean ± SD of three independent experiments. ^*^P < 0.05 versus vehicle-treated control.

To examine the antiangiogenic potential of MP0250 *in vivo*, we first performed the CAM assay. Addition of MP0250 to medium supplemented with VEGF and HGF or to RPMI8266 cell conditioned medium (CM), which is rich of these angiogenic factors, [5] resulted in a strong reduction of vessel counts around the sponges (Figure 4A) by approximately 60% (Figure 4B). MP0250 alone in the absence of added VEGF and HGF showed no significant effect in the model (Figure 4B).

Next, we performed the Matrigel plug assay by subcutaneously injecting NOD/SCID mice with a solution of Matrigel supplemented with VEGF and HGF or with RPMI8266 CM. Plugs obtained from the untreated and vehicle-treated groups were more vascularized, as confirmed by their red color. In contrast, plugs removed from MP0250-treated mice showed macroscopically inhibition of vascularization by their lighter color similar to the negative control (Matrigel alone) (Figure 4C). In addition, Figure 4D shows a significantly lower hemoglobin concentration in plugs from MP0250-treated mice compared to the control group. Data indicate that MP0250 significantly inhibits angiogenesis *in vivo*.

### MP0250 impacts angiogenesis in syngeneic 5T33MM model

**Figure 5:**
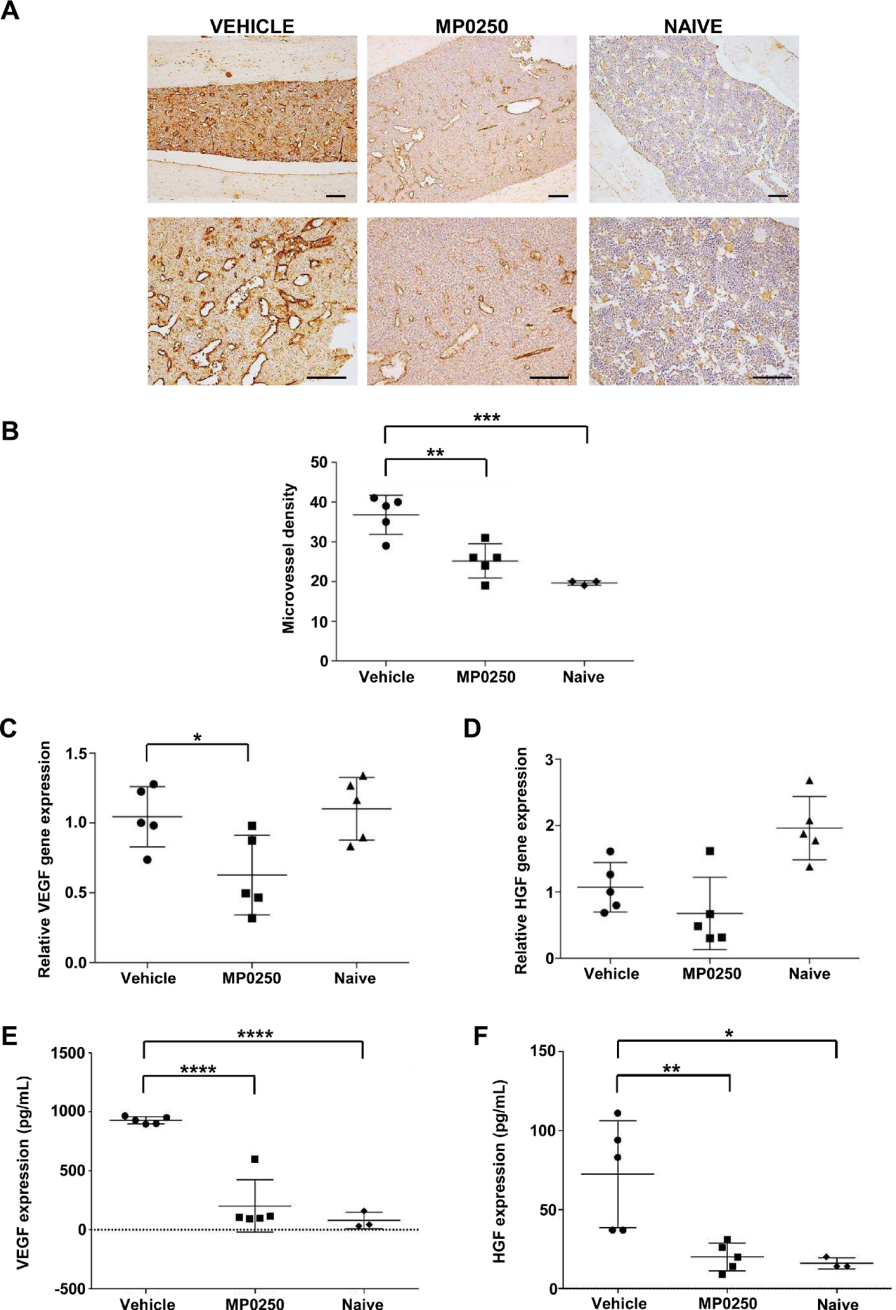
MP0250 reduces angiogenesis in 5T33MM mice C57BL/KaLwRij mice were inoculated with 5T33MM cells and at day 3, mice were treated with vehicle or MP0250 (4 mg/kg, every third day, intraperitoneally) for 21 days (*n* = 5/group). (**A**) MVD was analyzed by CD31 staining on BM sections. A representative picture for each group is shown (original magnification X100, X200). (**B**) Blood vessels were counted in a 0.22 mm^2^ area. (**C**–**D**) Total BM was isolated from untreated and MP0250-treated mice and analyzed for VEGF-A and HGF mRNA expression by qRT-PCR. (**E**–**F**) Plasma was collected and VEGF-A and HGF protein levels were determined by ELISA. Values are expressed as mean ± SD. ^*^P < 0.05, ^**^P <0.01, ^***^P < 0.001, ^****^P < 0.0001 versus control.

In the next set of experiments, we investigated whether MP0250 affects angiogenesis in the syngeneic 5T33MM mouse model. Previously it was demonstrated that 5T33MM murine myeloma cells and inflammatory cells in the mouse BM microenvironment secrete angiogenic factors such as VEGF and HGF [20, 21]. MP0250 is able to bind and neutralize murine VEGF and HGF [22], and was therefore tested in this model. Mice were inoculated with 5T33MM cells and, starting on day 3, were treated with MP0250 every 3 days for 21 days. MVD was analyzed by CD31 staining showing a significant reduction in angiogenesis *in vivo* after treatment with MP0250 (Figure 5A–5B). Furthermore, we isolated BMMC from naïve, untreated and MP0250-treated mice and observed a reduction in VEGF mRNA levels upon treatment, while HGF was unaffected (Figure 5C–5D). Interestingly, plasma VEGF and HGF levels were both significantly decreased in MP0250-treated mice compared to untreated mice, and even reached levels comparable to those of naïve mice, indicating that MP0250 is efficiently neutralizing these angiogenic factors in the model (Figure 5E–5F). Data demonstrate that MP0250 modifies the BM microenvironment by modulating VEGF and HGF secretion and neutralization, and thereby restrains angiogenesis.

### MP0250 improves bortezomib activity in MM

**Figure 6:**
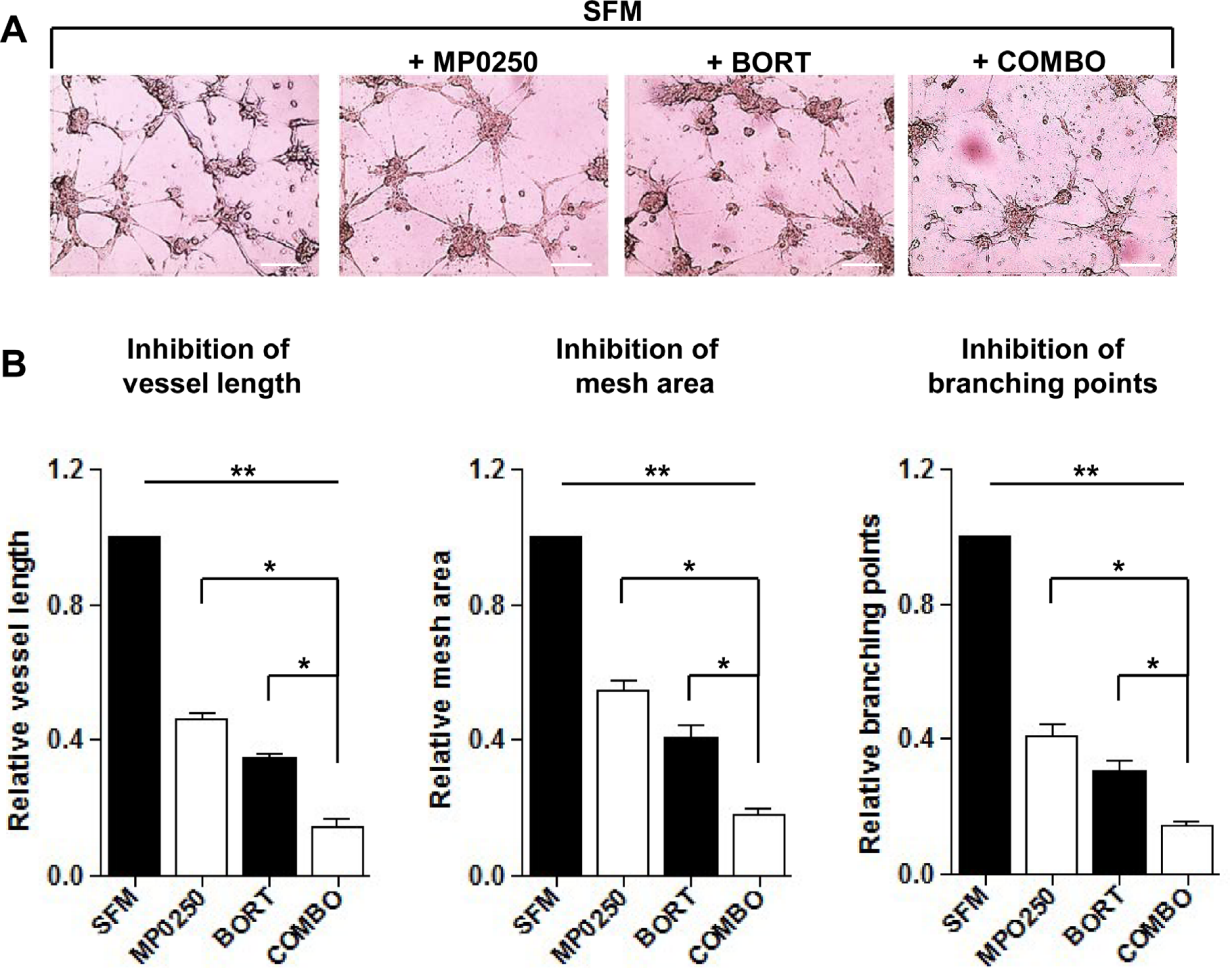
*In vitro* MP0250 synergism with bortezomib on Matrigel angiogenesis assay (**A**) MP0250 potentiates the antiangiogenic effect of bortezomib. MMEC were plated on Matrigel layer in SFM alone or added with bortezomib (5 nM), or MP0250 (2 µM) or with their combination. After overnight incubation representative pictures were taken. Original magnification X200; scale bar = 50 µm. (**B**) Normalized bar graphs confirm the effect of drugs combination. Quantitative analysis of vessels length, mesh areas and number of branching points was performed. Statistical significances are expressed as mean ± SD of ten experiments ^*^P < 0.05; and ^**^P < 0.01 versus untreated control.

Bortezomib is a proteasome inhibitor and the most commonly used drug in MM. Besides its direct effect on MM cells, bortezomib modulates the BM microenvironment and has antiangiogenic properties [11]. We wondered whether MP0250 could further increase the antiangiogenic and anti-MM activity of bortezomib. An *in vitro* Matrigel assay using MMEC treated with MP0250 or bortezomib alone showed that both drugs have antiangiogenic effects, and that their combination provided an additional decrease in angiogenesis (Figure 6A). Vessel area, branching points and vessel length were strongly reduced by the combo treatment (Figure 6B).

**Figure 7:**
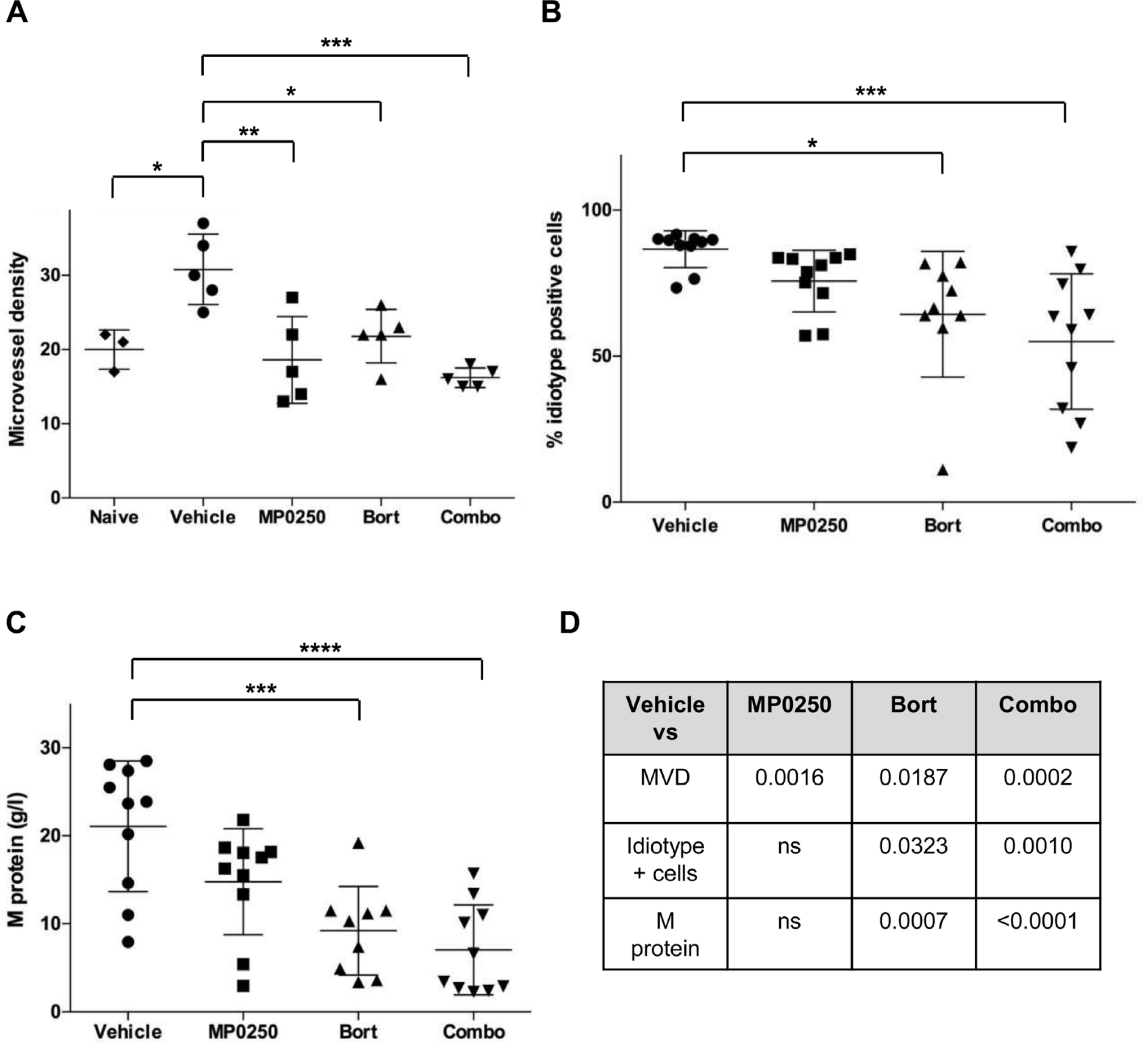
MP0250 sustains bortezomib activity in MM C57BL/KaLwRij mice were inoculated with 5T33MM cells and at day 3 mice were treated with MP0250 (4 mg/kg, every third day, intraperitoneally), bortezomib (Bort) (0.6 mg/kg, twice a week, subcutaneously) or the combination (Combo) for 21 days. (**A**) MVD was analyzed by CD31 staining on BM sections (*n* = 5/group). (**B**) Tumor load in BM by flow cytometry for idiotype staining (*n* = 10/group). (**C**) Serum M protein was analyzed by electrophoresis (*n* = 10/group). (**D**) Schematic summary of *P*-values. Values are expressed as mean ± SD. ^*^P < 0.05, ^**^P < 0.01, ^***^P < 0.001, ^****^P < 0.0001 versus control.

Furthermore, we investigated the combo treatment in the 5T33MM model. BM sections from MP0250-, bortezomib- and combo-treated mice revealed a significant reduction of MVD compared to vehicle-treated mice. (Figure 7A). Bortezomib- and combo-treated mice had also lower percentages of BM idiotype positive cells and lower levels of serum M protein. Notably, the higher statistical significance was achieved comparing the vehicle group to the combo-treated one. Negligible differences were instead found between bortezomib- and combo-treated mice (Figure 7B–7D).

### MP0250 affects MMEC secretory profile

**Figure 8:**
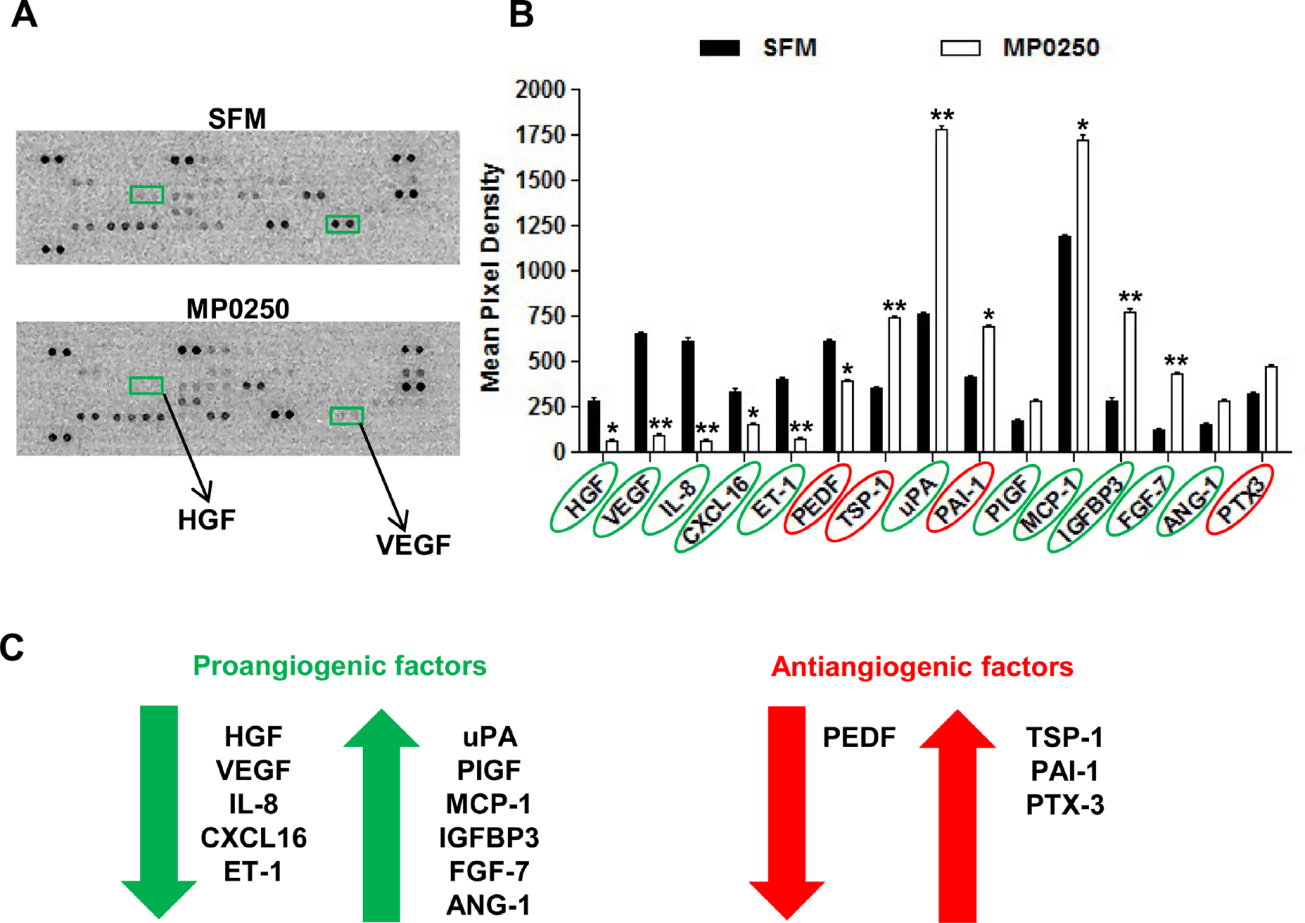
MP0250 modulates the secretory profile of MMEC (**A**) MMEC were treated with 2 µM MP0250 and with SFM, and media were collected and analyzed using a human angiogenesis array kit. Representative membranes are shown. VEGF and HGF spots were highlighted (green square). (**B**) Graph of quantitative results for angiogenesis protein arrays. Values are expressed as mean ± SD of six independent experiments. ^*^P < 0.05, ^**^P < 0.01. (**C**) MP0250 modulation of angiogenic and antiangiogenic factors is reported.

The effect of MP0250 on MMEC secretion of angiogenesis-related proteins was analyzed using a human angiogenesis array. Figure 8A shows MP0250 efficacy highlighting that VEGF and HGF spots had almost disappeared in the treatment array (green square). MP0250 modulated the expression of both angiogenic and antiangiogenic factors (Figure 8B). Among the angiogenic group, VEGF, HGF, interleukin-8 (IL-8), C-X-C motif chemokine ligand 16 (CXCL16) and endothelin-1 (ET-1) were decreased. MP0250 increased the expression of urokinase-type plasminogen activator (uPA), placental growth factor (PIGF), monocyte chemoattractant protein-1 (MCP-1), insulin-like growth factor-binding protein 3 (IGFBP3), fibroblast growth factor-7 (FGF-7) and angiopoietin 1 (ANG-1). Thrombospondin-1 (TSP-1), plasminogen activator inhibitor-1 (PAI-1), pentraxin-3 (PTX-3) were the only up-regulated antiangiogenic factors, while pigment epithelium-derived factor (PEDF) expression was decreased (Figure 8C). Therefore, MP0250 alters the balance between pro- and antiangiogenic factors, and as functionally observed *in vitro* and *in vivo,* it results in an overall antiangiogenic balance.

## DISCUSSION

MP0250 is a tri-specific DARPin^®^ molecule that binds to VEGF, HGF and human serum albumin, giving simultaneous neutralization of the growth factors and binding to albumin gives the molecule an increased duration of action *in vivo*. The molecule has demonstrated anti-tumor and antiangiogenic effects in multiple preclinical *in vivo* studies [18] , and demonstrated a good safety profile in Phase I clinical trial in advanced solid tumors [17, 23]. In this study the antiangiogenic potential of MP0250 was tested in various MM models. The aim of envisaging a molecule able to specifically recognize VEGF and HGF that are key angiogenic factors was the creation of an innovative tool halting complex processes such as tumor angiogenesis [6]. In MM, the involvement of both of the main growth factors VEGF and HGF is well recognized. MMEC are characterized by a peculiar activated phenotype sustained mostly by the VEGF autocrine loop. Its constitutively activated downstream signaling stimulates all the angiogenic functions involved in the new vessel formation in the BM [19]. Moreover, the role of the HGF/cMet axis in MM angiogenesis and progression has been demonstrated [8]. The cooperation between VEGF and HGF was highlighted showing that in ECs they amplify the activation of various signaling kinases [24]. Functionally their synergism promotes *in vitro* ECs survival, migration and tubulogenesis in 3-D type I collagen gels as well as *in vivo* neovascularization using rat corneal assay [25].

Here we demonstrate that MP0250 acts as an antiangiogenic agent in MM, both *in vitro* and *in vivo.* We found that MP0250 exerts a rapid concentration-dependent effect on VEGFR2 and cMet activation. It affects also the phosphorylation of ERK1/2, p38 MAPK, AKT and PLCγ1 that are intracellular mediators involved in the over-angiogenic phenotype of MMEC [19]. These reduced levels of protein phosphorylation were observed to be persistent up to 12 hours that explains the impairment of MMEC angiogenic functions.

MP0250 strongly alters spontaneous and chemo-induced MMEC migration and cell spreading capacity while adhesion is not significantly impacted. Conceivably, this may be due to intracrine growth factor stimulation that may keep the downstream signaling cascades that governs the adhesion function [26]. However, the *in vitro* Matrigel assay testing the complete angiogenesis process confirms that MP0250 is able to alter the structure of the capillary network by the neutralization of the autocrine VEGF and HGF.

In the MM microenvironment, several cell types contribute to angiogenesis by releasing VEGF and HGF that activate MMEC [3]. To evaluate the antiangiogenic properties of MP0250 under conditions of boosted angiogenesis, we performed the *in vitro* Matrigel angiogenesis assay in the presence of exogenous VEGF and HGF. MP0250 inhibits vessel formation more profoundly compared to VEGF or HGF neutralizing mAbs, thus confirming the potential of the simultaneous inhibition of each pathway. To further assess the potential of MP0250 in complex systems we performed an *in vivo* CAM assay and a Matrigel plug assay. To boost the angiogenic process we injected a solution containing both VEGF and HGF or the CM of MM cells. The results showed that in these conditions MP0250 is able to strongly neutralize VEGF and HGF causing a significant reduction in the vessel number. Profiling for angiogenesis related cytokines of MMEC treated with MP0250 confirmed that neutralization of VEGF and HGF results in a change of the cytokine profile towards in favor of antiangiogenic acting cytokines.

To validate the antiangiogenic potential of MP0250 *in vivo* we exploited the 5T33MM syngeneic preclinical model. This model recapitulates all the features of human MM including increased angiogenesis in the BM during the disease progression [20, 27]. Treatment with MP0250 reduced MVD down to the values of the control group. Moreover, plasma levels of both VEGF and HGF were similar to levels obtained in naïve mice. It is plausible to hypothesize that MP0250 may be clinically beneficial even in those MM patients with advanced disease accompanied by increased levels of VEGF and HGF [28, 29].

Considering that in clinical practice antiangiogenic drugs are combined with other anti-MM agents including chemotherapy and proteasome inhibitors, we administered MP0250 with bortezomib in the 5T33MM model. Tumor load was highly reduced after combo treatment with bortezomib and MP0250 compared to vehicle group. On the contrary, no tumor shrinkage was obtained when MP0250 was used alone. This result contrasts with the recent observation that MP0250 owns direct anti-tumor activity and enhances the efficacy of several chemotherapy drugs in different tumor models [18]. The reasons underlying this discrepancy remains unclear. Nevertheless, the finding that microvessel density, percentage of idiotype positive cells, and serum M-protein levels, all tended to be lower in the combo group compared to bortezomib group encourages further investigation. Additional studies are also required to define the role of MP0250 in MM immunotherapy protocols, as soon as promising results have been obtained by its combination with an anti-PD-1 molecule in a colon carcinoma syngeneic model [18].

In conclusion, our data indicate that MP0250 displays antiangiogenic properties in various MM models. Common antiangiogenic strategies targeting a single factor may result in the activation of alternative compensatory pathways responsible for the resistance to antiangiogenic treatments [30, 31]. MP0250 represents an innovative approach in MM thanks to its dual neutralization of VEGF and HGF that allows the inhibition of common pathways found activated in resistant patients after treatment with conventional therapy. Given that HGF is a well-known prognostic factor for MM progression and acts as pro-survival cytokine for MM tumor cells [32], it is likely that in human clinical trials HGF neutralization will add further anti-proliferative potential to MP0250 antiangiogenic effect. To prove these hypothesis, a Phase II clinical trial will start enrolling patients with refractory and relapsed MM, who have received ≥2 lines of therapy (including bortezomib and IMIDs). The trial will evaluate MP0250 in combination with bortezomib and dexamethasone (Clinical trial Identifier: NCT03136653). Given the promising preclinical results, clinical data from the above-mentioned Phase II study will hopefully confirm MP0250 as new antiangiogenic drug for MM, that it is still an incurable disease for the majority of patients.

## MATERIALS AND METHODS

### Patients

Twenty patients fulfilling the International Myeloma Working Group diagnostic criteria for active MM [33] were studied. They were 14 men and 6 women, age range 50 to 78 (median 67) years, and staged according to Durie & Salmon. The study was approved by the Ethical Committee of the University of Bari Medical School. All patients provided their written informed consent in accordance with the Declaration of Helsinki.

### Reagents

MP0250 (Molecular Partners AG, Zürich, SW) had a concentration of 15 mg/mL (220 µM) and was stepwise diluted in culture medium before use. Bortezomib (Velcade^®^, Janssen-Cilag) was dissolved at 5 nM in PBS. Rabbit anti-VEGF-A 165 and anti-HGF antibodies were respectively purchased from R&D System (Abingdon, UK); recombinant human HGF and human VEGF-A 165 from Miltenyi Biotec (Bergisch Gladbach, Germany); Matrigel™ Basement Membrane Matrix from Becton Dickinson-BD Bioscience (San Jose, CA, USA).

#### Cell separation and cultures procedures.

MMEC were obtained by centrifugation on Ficoll-Hypaque gradient of heparinized bone marrow (BM) aspirates. MMEC were isolated from BM mononuclear cells (BMMC) using anti-CD31 MACS beads (Miltenyi Biotec, Bergisch Gladbach, Germany) and grown in Dulbecco’s modified Eagle’s medium (DMEM) supplemented with 20% heat-inactivated fetal bovine serum (FBS) [34]. Cell population purity (>95%) was determined using the FACScantoII flow cytometry system (Becton Dickinson-BD, San Jose, CA, USA). In functional studies, MMEC were used until the 7th passage of culture. Human RPMI8266 MM cells were obtained from the American Type Culture Collection (ATCC; Rockville, MD, USA), maintained in Roswell Park Memorial Institute medium (RPMI)-1640 supplemented with 10% FBS and routinely tested for mycoplasma contamination. To obtain conditioned media (CM), RPMI8266 cells were grown to 80% confluence in serum-free RPMI-1640 medium for 48 hours. Culture media, antibiotic/antimycotic, glutamine, trypsin/EDTA, and PBS without Ca^2+^ and Mg^2+^ were all purchased from Sigma-Aldrich (St Louis, MN, USA).

### Functional studies

#### Cell viability

It was determined using the CellTiter-Glo^®^ Luminescent Cell Viability Assay (Promega, Madison, WI, USA) according to the manufacturer’s instructions.

#### Apoptosis

It was quantified as previously described [35] by AnnexinV-PE/7aminoactinomycin-D staining (BD), followed by flow cytometric analysis. Samples were acquired on the FACSCantoII flow cytofluorimeter.

#### Chemotaxis, “wound” healing”, spreading, adhesion and Matrigel angiogenesis assay

Assays were performed as previously described [36, 37].

#### Adhesion assay

MMEC (10 × 10^3^) were labeled with Calcein-AM (5 µM, Molecular Probes, Eugene, OR, USA), plated in triplicate on 96 well-plates, and incubated in SFM with or without MP0250 2 µM for 30 minutes. Data were quantified on a microplate reader (PerkinElmer VICTOR X, Singapore) [36, 37].

#### Spreading assays

MMEC were plated (5 × 10^3^ cells per well) in triplicate in fibronectin-coated (10 mg/ mL) 96 well-plates in SFM alone or with MP0250 2 µM for 90 minutes. Then cells were fixed with 4% paraformaldehyde, stained with crystal violet, and quantified at 595 nm using the VICTOR X reader [36, 37]

#### Chemotaxis

MMEC (6 × 10^4^ cells per well) were seeded on the upper compartment on a micropore nitrocellulose filter (8 µm pores) coated with fibronectin (Sigma Aldrich). MMEC migration toward SFM alone (negative control) or SFM supplemented with HGF and VEGF-A 165 (50 ng/mL for each growth factor) or with 2 μM MP0250 was evaluated after 6 hours at 37° C. MMEC were counted at X400 using EVOS inverted microscope (Euroclone, Milan, Italy).

#### “Wound” healing” assay

ECs were grown until confluence on fibronectin-coated (10 mg/mL) 24 well plate and the “wound” was made by scraping the cell monolayer with a P200 pipette tip. Cells were exposed for 24 hours to SFM alone (negative control) or admixed with MP0250 2 µM. The migrating MMEC were counted into 3 different fields of the wound area of each X10 field with an EVOS digital inverted microscope (Euroclone).

#### Matrigel angiogenesis assay

MMEC were plated on Matrigel-coated (BD) 24-well plates in SFM alone (positive control), in SFM supplemented with VEGF-A and HGF (50 ng/mL for each growth factor), in presence or absence of MP0250 2 µM. MMEC were also plated in presence of anti-VEGF neutralizing/blocking mAb (cod. MAB293) 0.06 μg/mL and anti-HGF neutralizing/blocking mAb (cod. MAB294) 0.2 μg/mL (R&D system, Minneapolis, MN, USA). After 18 hours, pictures of the skeletonized mesh were acquired with EVOS microscope. The topological parameters (“mesh areas,” “length,” and “branching points”) were measured with a computerized image analyzer.

#### Immunofluorescence

Immunofluorescence on MMEC was performed as previously described [38]. MMEC were plated on chamber slides and spreading assay was performed as described in Material and Methods. After that MMEC were washed with PBS, fixed in paraformaldehyde for 15 minutes, permeabilized with 0.2% Triton X-100 for 30 minutes, blocked with 3% bovine serum albumin for 60 minutes, and then incubated with anti-phalloidin antibody (Sigma Aldrich). Nuclei were counterstained with 4′,6-diamidino-2-phenylindole (Molecular Probes, Eugene, OR, USA). Slides were examined using Olympus fluorescence Microscope (Olympus Italia, Rozzano, Italy).

#### Study of VEGFR2 and cMet phosphorylation

It was evaluated by flow cytometry and events/sample were acquired and analyzed using FACScantoII flow cytofluorimeter. MMEC were incubated overnight with or without MP0250 (ranging from 0.4 µM to 3.2 µM) in SFM, fixed with 2% paraformaldehyde, permeabilized in cold 90% MeOH and immunostained with rabbit IgG anti-human VEGFR2 and rabbit mAb to phospho-cMet (R&D System) followed by staining with PE-labeled anti-rabbit IgG antibody (BD). At least 100,000 events/sample were acquired and analyzed using the FACScantoII flow cytofluorimeter. Negative controls were stained with irrelevant isotype antibodies (BD).

#### Human angiogenesis array

MMEC were treated with 2 µM MP0250 and medium was collected and concentrated to be analyzed on the Human Angiogenesis Array kit (R&D System) according to the manufacturer’s instructions. Spots were quantified with Kodak Molecular Imaging Software (Rochester, NY, USA) and values were reported as mean pixel density.

#### Western blot

Total MMEC protein lysates were quantified with the Bradford assay (Bio-Rad, Hercules, CA, USA) and subjected to immunoblot with primary and secondary antibodies to the following: cMet (cod.#8198), phospho (p)-cMet (cod.#3077), VEGFR2 (cod.#9698), p-VEGFR2 (cod.#3770), p-extracellular signal regulated kinase (p-Erk)-1/2 (cod.#4370), Erk-1/2 (cod.#9102), p-Protein kinase B (p-AKT) (cod.#4060), AKT (cod.#9272), p-Phosphoinositide-specific phospholipase C (p-PLC)γ1 (cod.#8713), PLCγ1(cod.#5690), p-P38 mitogen-activated protein kinases (p-p38) (cod.#4511), p38 (cod. #8690) (Cell Signaling Technology, Danvers, MA, USA); beta-actin (Sigma-Aldrich) (cod. A1978); and mouse and rabbit horseradish peroxidase–conjugated IgG (Bio-Rad). Immunoreactive bands were visualized by enhanced chemiluminescence (SuperSignal West Femto Maximum Sensitivity Substrate, Thermo Scientific, Waltham, MA, USA) and the Gel Logic 1500 Imaging System (Eastman Kodak Co.), quantified with the Kodak Molecular Imaging Software, and expressed as arbitrary optical density (OD).

### *In vivo* angiogenesis

#### CAM assay and matrigel plug assay

They were performed as previously described [38, 39]. Fertilized white Leghorn chicken eggs were incubated at 37° C at constant humidity. On day 3, the shell was opened and 2 to 3 mL of albumen was removed to detach the chorioallantoic membrane (CAM). On day 8, the CAMs were implanted with 1 mm^3^ sterilized gelatin sponges (Gelfoam, Upjohn Co, MI, USA) filled with serum free medium (SFM) alone (negative control) or with 20 μM of MP0250, or SFM supplemented with 50 ng/mL of HGF and VEGF (positive control) in presence or absence of 20 μM of MP0250 and with RPMI8266 MM cells CM alone or with MP0250. On day 12, the vessels converging toward the sponge were counted and pictures taken *in ovo* at X50 (Olympus stereomicroscope).

#### Matrigel plug assay

Six to eight week-old non-obese diabetic (NOD) severe combined immunodeficiency (SCID) NOD.CB17-Prkdcscid/NCrHsd mice (Envigo, Huntingdon, UK) were subcutaneously injected with a mixture containing growth factors reduced Matrigel and heparin (50 U/mL) supplemented with HGF and VEGF (200 ng/mL for each growth factor) or with RPMI8266 cell (5x10^5^ cells/mL) CM. Assuming an effect-size of 0.4 with statistical significance of α < 0.05 and a power of 80% we used n. 6 mice for each group for a total of 64 mice. This number was increased to 72 taking into account an expected drop-out rate of 10% for the treatment group. Drop-outs, specifically, may be caused by failure of plug injection. Mice were administered intraperitoneally every 72 hours with MP0250 (4 mg/Kg) or with PBS. After 14 days, mice were sacrificed, and the Matrigel plugs removed, weighed, and homogenized in cold PBS. The hemoglobin content of the plugs was calculated using Drabkin’s reagent kit 525 (Sigma-Aldrich) and normalized to the weights. Mice were housed according to the Institutional Animal Care and Use Committee of the University of Bari Medical School, Bari, Italy (licence n. 548/2016PR).

#### 5T33MM syngeneic mouse model

Seven weeks old male C57BL/KaLwRij mice (Harlan CPB, Horst, the Netherlands) were housed and maintained following the conditions approved by the Ethical Committee for Animal Experiments, Vrije Universiteit Brussel (license no. LA1230281, 16-281-2). Assuming an effect-size of 0.4 with statistical significance of α <0.05 and a power of 80% for the *in vivo* experiment, we used n. 9 mice for each group for a total of 72 mice. This number was increased to 80 taking into account an expected drop-out rate of 10% early mortality. Drop-outs, specifically, may be caused by failure of tumor uptake (for intravenous injection).

MVD was determined by CD31 staining on BM sections. A femur was fixed in zinc fixative for 48 hours, decalcified for 48 hours and paraffin embedded. Paraffin sections were blocked with normal goat serum and incubated with a rat anti-CD31 antibody (PECAM-1, 1:10; PharMingen, San Diego, CA, USA) overnight at 4° C. A biotin-conjugated goat anti-rat antibody was used as a secondary antibody (1:75; BD). A streptavidin-horseradish peroxidase conjugate in combination with tyramide signal amplification (TSA; NEN Life Science Products, Boston, MA, USA) was used for detection. The number of blood vessels in the area with the highest blood vessel density was counted per 0.22 mm^2^.

Plasma was collected and analyzed for VEGF-A (R&D System) and HGF (Thermo Scientific, Aalst, Belgium) expression using a 1/2 dilution.

BM cells were isolated from one leg, followed by red blood cell lysis. RNA was extracted by the RNeasy mini kit (Qiagen, Venlo, The Netherlands) and converted into cDNA using the First-strand cDNA synthesis kit (VWR International, Leuven, Belgium). Expression levels of VEGF and HGF mRNA were quantified by qRT-PCR using ABI 7900TH Real-Time PCR System (Applied Biosystems, Foster City, CA, USA). Primer sequences are: VEGF forward primer CACTTCCAGAAACACGACAAAC, VEGF reverse primer TGGAACCGGCATCTTTATCTC, HGF forward primer ATCCCAAATCGT CCTGGTATTT and HGF reverse primer CTGGCCTCTTCTATGGCTATTAC.

Tumor load in the 5T33MM model was analyzed by the use of anti-idiotype (3H2) mAb (IgG1) and APC-labeled rat anti-mouse IgG1 Ab (secondary step) [40].

### Statistical analysis

Results are expressed as individual data or as the mean ± SD and were analyzed using Wilcoxon signed-rank test, Mann-Whitney *U* test and One-Way Anova test. *P* < 0.05 was considered statistically significant. Statistical analysis was performed using GraphPad Prism 5 software (La Jolla, CA, USA). For *in vivo* experiments, sample size was calculated using G^*^Power software version 3.1.9.2 (power of 80 % and 0.05 statistical level).

## SUPPLEMENTARY MATERIALS FIGURES



## Abbreviations

DARPin: designed ankyrin repeat proteins
MM: multiple myeloma
EC: endothelial cells
BM: bone marrow
VEGF: vascular endothelial growth factor
HGF: hepatocyte growth factor
VEGFR2: vascular endothelial growth factor receptor 2
cMet: hepatocyte growth factor receptor
CAM: choriollantoic membrane assay
MVD: microvessel density
PC: plasma cells
mAb: monoclonal antibody
IMiDs: immunomodulatory drugs
