# Supplementary material for: Targeting angiogenesis in multiple myeloma by the VEGF and HGF blocking DARPin® protein MP0250: a preclinical study
Source: Oncotarget. 2018 Jan 30;9(17):13366–81. doi: 10.18632/oncotarget.24351 (PMC5862584; doi:10.18632/oncotarget.24351)
Supplement: Supplementary file 1 [file oncotarget-09-13366-s001.pdf]

Targeting angiogenesis in multiple myeloma by the VEGF and HGF blocking darpin® protein MP0250: a preclinical study

SUPPLEMENTARY MATERIALS

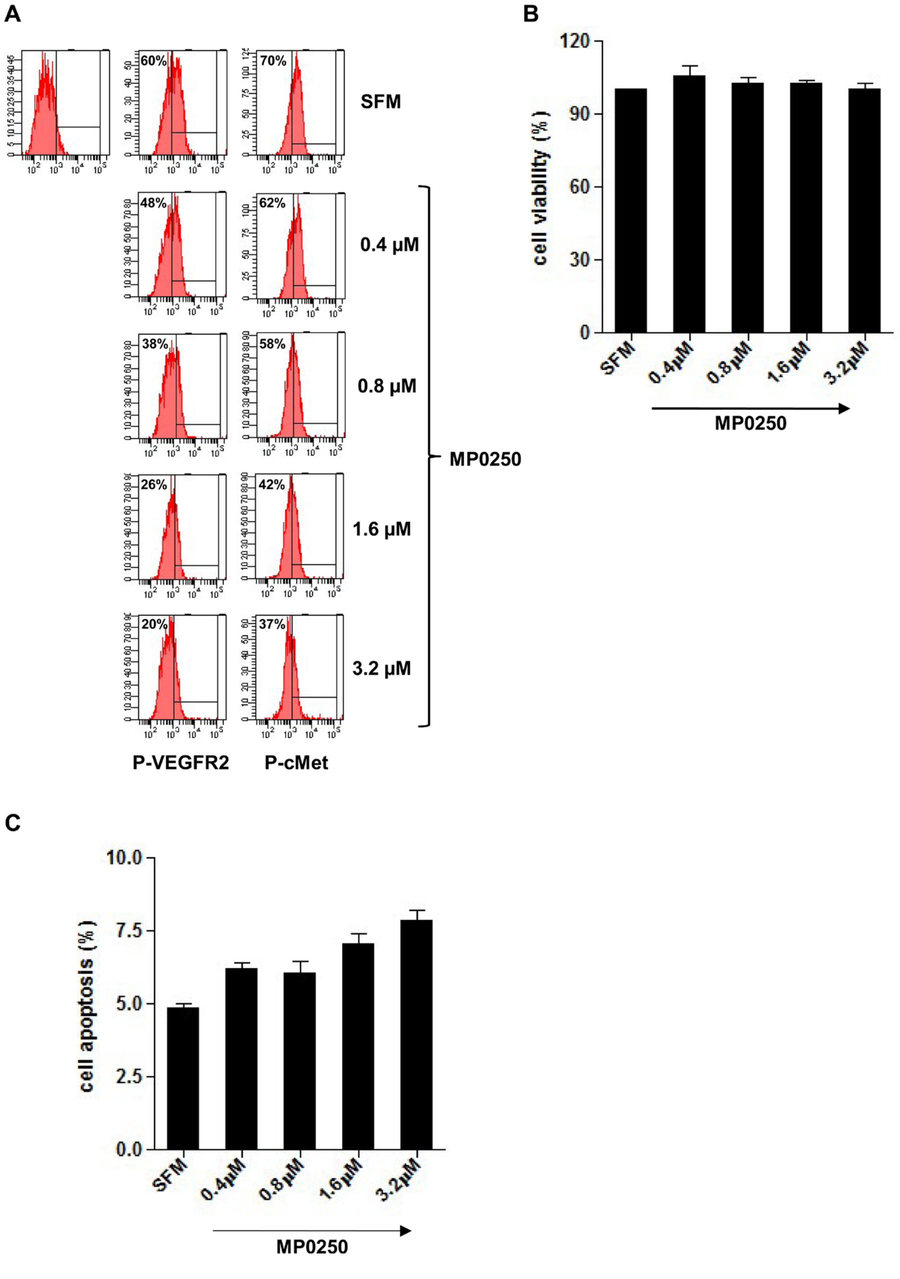

Supplementary Figure 1: Dose-dependent effect of MP0250 on VEGFR2 and cMet phosphorylation (p), cell viability and apoptosis. (**A**) p-VEGFR2 and p-cMet expression was evaluated on MMEC treated with SFM or increasing doses of MP0250 (range 0.4–3.2 µM) for 12 hours. A representative cytofluorimetric analysis of ten experiments is shown. MMEC were cultured in SFM for 24 hours in presence of MP0250 at increasing concentration (range 0.4–3.2 µM). (**B**) Viability was measured by CellTiter-gloo Luminescent Cell Viability Assay. Results are reported as percentage of cell viability using untreated cells as control. (**C**) Simultaneously, apoptosis was evaluated by flow cytometry by Annexin-V-PE/7-AAD staining. Representative experiments of ten are shown. Each experiment was done in triplicate.

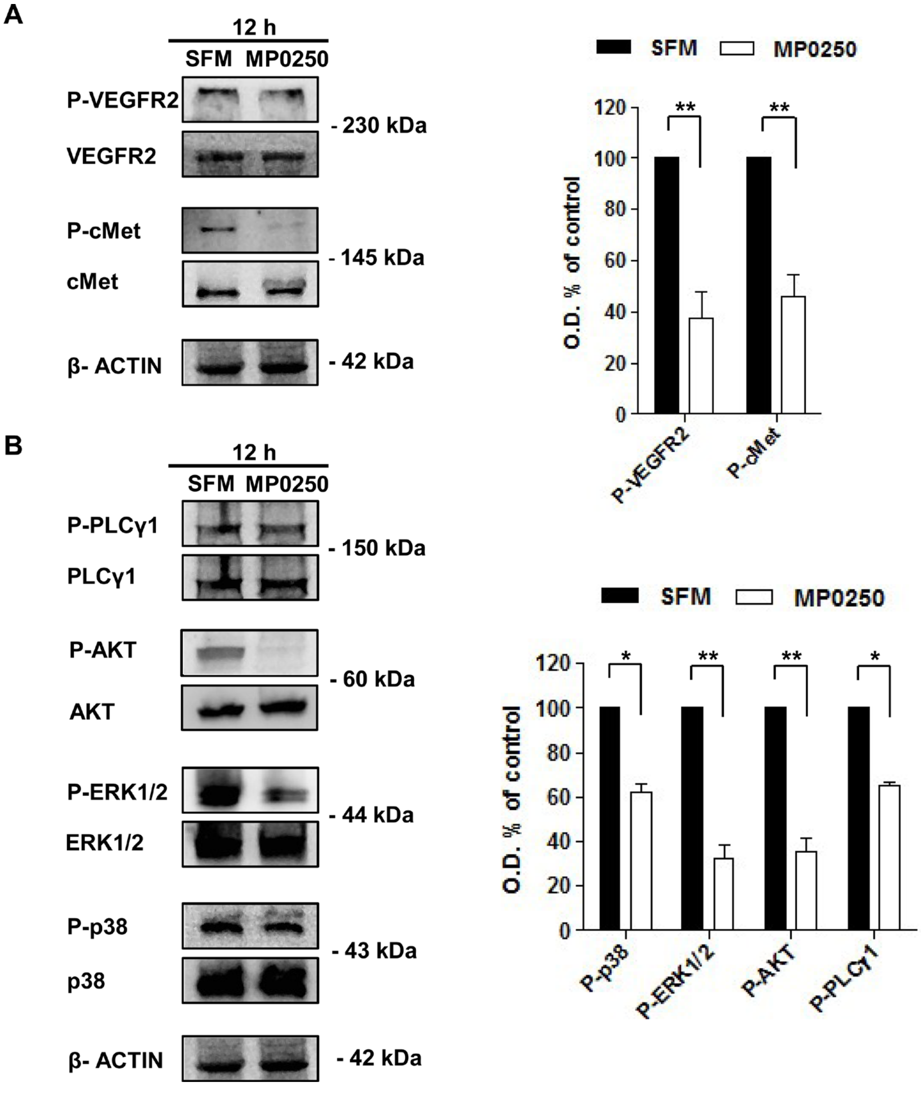

Supplementary Figure 2: Overnight treatment of MMEC with MP0250 shows persistent modulation of p-VEGFR2 and p-cMet, and their signaling cascade. (**A**) MMEC were incubated for 12 hours with MP0250 2 μM and lysates were immunoblotted to evaluate p-VEGFR2 and p-cMet. A representative western blot panel is reported. Graph shows the results obtained by six independent experiments. (**B**) Phosphorylation levels of AKT (Ser473), PLCγ1 (Ser1248), P38 (Thr180/Tyr182) and ERK1/2 (Thr202/Tyr202) were analyzed. The panel shows representative results from six independent experiments. Densitometric analysis is shown. Statistical significances are expressed as mean ± SD, $^{*}P < 0.05$; and $^{**}P < 0.01$ versus SFM as control.
